# Supplementary figures and images for: Microglial-stimulation of glioma invasion involves the EGFR ligand amphiregulin
Source: PLoS One. 2021 Nov 29;16(11):e0260252. doi: 10.1371/journal.pone.0260252 (PMC8629255; doi:10.1371/journal.pone.0260252)

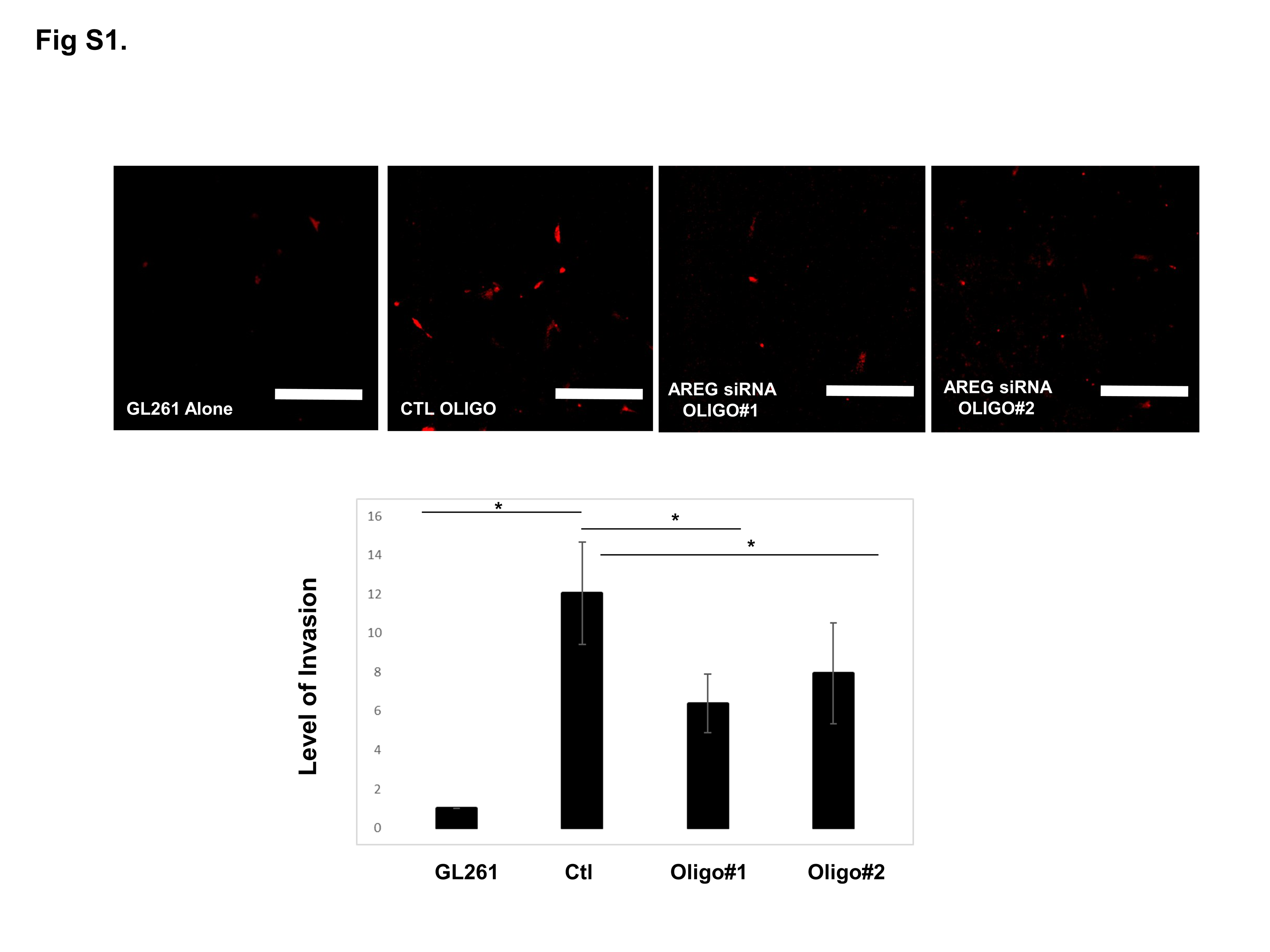

Supplement: S1 Fig — Microglial cells depleted with either control or AREG siRNA individual oligos were cocultured with GL261 cells expressing mCherry on Matrigel-coated invasion chambers. Representative images are shown. Arrows indicate fluorescently labeled glioma cells which have invaded to the other side of the filter. Scale bar = 200 um. Results shown are average of at least five experiments. *: P < 0.05. (TIF) [file pone.0260252.s001.tif]

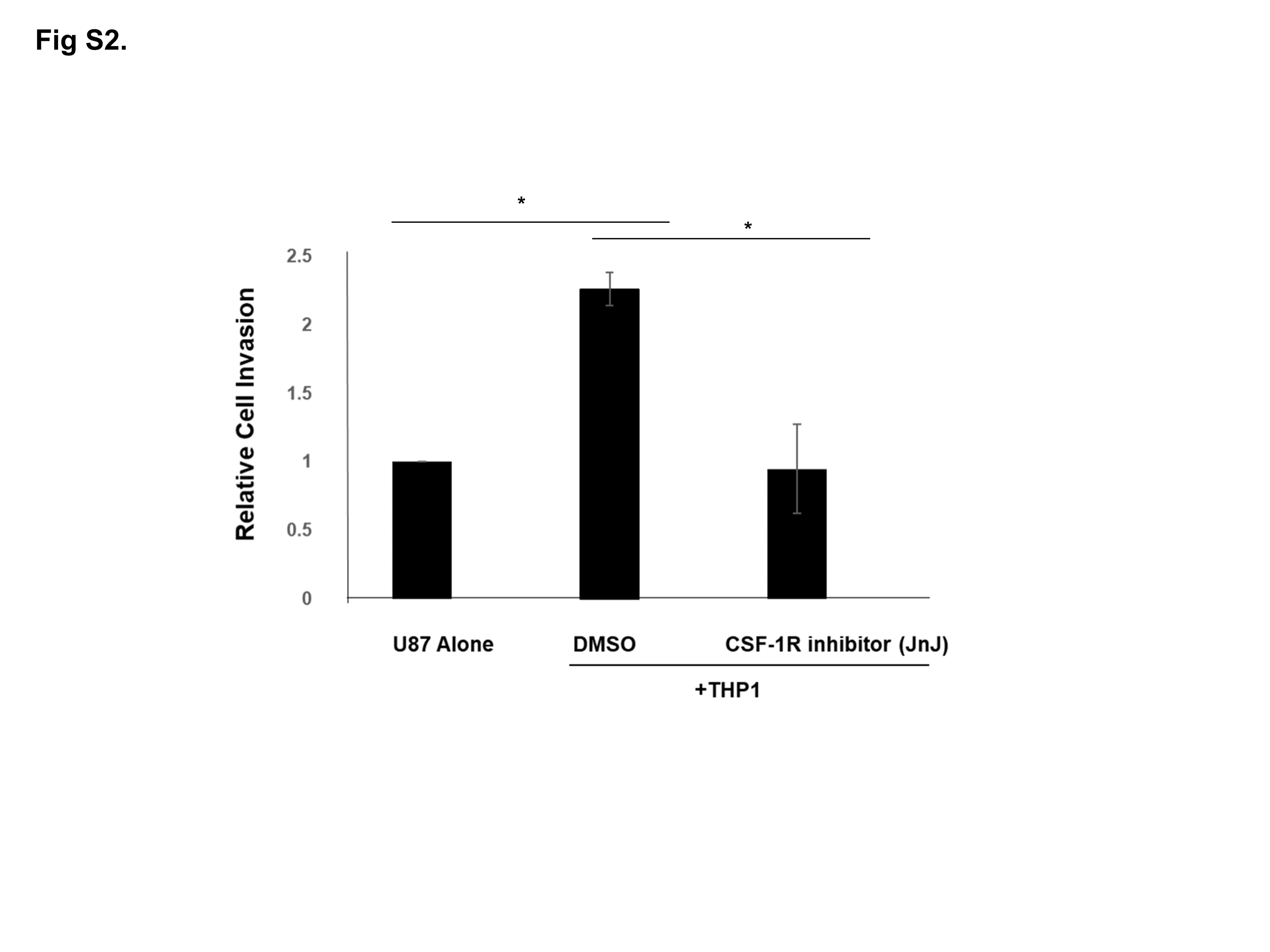

Supplement: S2 Fig — Results shown are average of at least three experiments. *: P < 0.05. (TIF) [file pone.0260252.s002.tif]
