# Supplementary material for: Microglial-stimulation of glioma invasion involves the EGFR ligand amphiregulin
Source: PLoS One. 2021 Nov 29;16(11):e0260252. doi: 10.1371/journal.pone.0260252 (PMC8629255; doi:10.1371/journal.pone.0260252)
Supplement: S1 Dataset — (PDF) [file pone.0260252.s003.pdf]

Fig 1A

EGF

	Untreated I	GLCM treated
	0.999	1.02
	8.1	0.69
	192	91
	81.7	33.5
	135.8	248.4
**Average:**	**83.7198**	**74.922**
SE	41.07814	51.86285956

HBEGF

	Untreated I	GLCM treated
	151.6	49.4
	25.6	406.8
	102.6	204.9
	122.2	17.2
	0.18	0.77
	18.3	13.7
**Average:**	**70.08**	**115.4616667**
SE	28.26023	72.22473239

AREG

	Untreated I	GLCM treated
	273.9	753.4
	554.8	3246.8
	21	1609.3
	47.7	660.1
	16.6	2168.1
	103.5	335.4
	117.9	774.5
	168.8	2054.4
	2	1.6
**Average:**	**145.1333**	**1289.288889**
SE	62.4251	370.9818902

EREG

	Untreated I	GLCM treated
	1231.3	8754.3
	15.3	1383.5

	10.6	307.7
	97.4	3360.231
	109.5	497.5
	0.9	226.9
	97.9	133.7
	48.7	1083.6
	10.8	92.2
	30.36896	157.2494325
	565.5386	1045.623194
	606.1293	1120.671191
	343.042	1392.431203
	259.3558	8421.261603
	644.9052	11845.14851
**Average:**	**271.4493**	**2654.801076**
SE	778	1013

TGFA

	Untreated I	GLCM treated
	1	63.8
	1	446.4
	1	532
	1	3.1
	1	27.8
	1	808.8
	1	359.6
	1	2.1
	1	112.1
	1	4.9
**Average:**	**1**	**236.06**
SE	0.005293	94.6819924

BTC

	Untreated I	GLCM treated
	183.5	284.2
	15.49119	19.07461897
	11.35984	70.65045761
	85.5535	402.3815465
	390.337	481.329043
**Average:**	**137.2483**	**251.5271332**
SE	78.87276	101.0512533

EPGN

	Untreated I	GLCM treated
	128.3	142

	22.5	590.5
	24.8	145.7
**Average:**	**58.53333**	**292.7333333**
SE	42.73825	182.3328145

Fig 1B

AREG mRNA (Fold increase over Microglia Alone control using 2^-(ddct

	Microglia al	Microglia Cocultured with GL261 Cells
	1	7.223483135
	1	4.481475056
	1	11.17752125
**Average:**	**1**	**7.627493148**
SE	0	1.94

Fig 2A

AREG mRNA (Fold increase over untreated control using 2^-(ddct))

Exp#	untreated	GLCM Treated	GLCM + CSF1R Inhibitor
1	1	2.738859007	1.467813
2	1	76.23833288	21.19228
3	1	13.83176796	1.29205
4	1	130.9202997	22.87494
5	1	5.137420115	3.792482
6	1	6.566510893	3.520777
7	1	9.724841029	7.557631
8	1	2.050135597	2.900915
9	1	7.622349006	3.728746
10	1	3.703185783	2.186648
11	1	13.90219711	5.803777
12	1	1.674905075	1.211871
13	1	9.359927359	2.52969
14	1	7.577631588	5.103229
15	1	4.812721698	4.871993
16	1	20.76782894	14.83394
17	1	9.217554235	11.36905
**Average:**	**1**	**19.16743929**	**6.83752**
SE:	0	8.391359957	1.692181

Fig 2B

AREG mRNA (Fold increase over untreated control using 2^-(ddct))

	untx	CSF1	GLCM
1	1		2.738859
2	1	1.99	11.16
3	1		76.23833
4	1		13.83177
5	1	5.89	130.9203
6	1	3.1	
7	1		5.13742
8	1		6.566511
9	1	5.06	12.2
10	1	1.6	
11	1		9.724841
12	1		2.050136
13	1		7.622349
14	1	5.53	3.703186
15	1		13.9022
16	1		1.674905
17	1		9.359927
18	1		7.577632
19	1		4.812722
20	1		20.76783
21	1		9.217554
**Average:**	**1**	**3.861666667**	**18.37929**
SE:	0	0.697851531	7.08615

Fig 2C

(Normalized to Actin and DMSO)

		GLCM (24 HRs)	
Exp#	DMSO	DMSO	JnJ
1	1	2.877	1.91
2	1	1.27	0.92
3	1	1.9	1.1
4	1	1.65	1.417
5	1	1.311783271	1.15241
**Average:**	**1**	**1.801756654**	**1.299882**
SE:	0	0.327076935	0.192349

Fig 3B

Exp#	w/GL	w/MG-CTLsiRI	w/MG-AREGsiRNA

1	3.4	11.95	5.266667
2	3.6	19.85	11.35
3	3.2	14	4.8
4	5.8	15.4	9.8
5	6.75	28.25	11.5
**Average:**	**4.55**	**17.89**	**8.543333**
SE:	0.950778	3.809782982	1.927482

Fig 4

(Normalized to U87 Alone invasion)

Exp#	U87 Alone	THP1 Control Ab	THP1 AREG Blocking Ab
1	1	2.666666667	2
2	1	5.1	3.35
3	1	3.76	1.04
4	1	12.5	2.5
5	1	6.107476636	5.079439
6	1	2.840136054	1.139456
7	1	10.08883249	5.34264
8	1	2.813688213	2.192015
9	1	2.423076923	2.051282
10	1	8	4.6
**Average:**	**1**	**5.629987698**	**2.929483**
SE:	0	1.173164045	0.527642

Fig 5B

AREG mRNA (Fold increase over untreated control using 2^-(ddct))

Exp#	untx	GLCM	U0126
1	1	5.137420115	2.017866
2	1	4.683232513	0.185815
3	1	3.818212331	0.864741
**Average:**	**1**	**4.54628832**	**1.022808**
SE:	0	0.478700717	0.661569

Fig 5C

(Normalized to CTL invasion)

Exp#	CTL	DMSO	U0126

1	1	6.052	3.8
2	1	4.08	1.76
3	1	2.888888889	1.666667
4	1	5	1.4
**Average:**	**1**	**4.505222222**	**2.156667**
SE:	0	0.777714387	0.639378

Fig S1

(Normalized to Ctl Oligo Invasion)

Exp#	**GL alone**	**Ctl Oligo**	**Oligo#1**	**Oligo#2**
1	0.184211	1	0.964912	
2	0.196226	1	0.566038	0.641509
3	0.229388	1	0.897959	0.346939
4	0.145978	1	0.497405	
5	0.047636	1	0.627273	
6	0.115385	1	0.813846	
7	0.067568	1	0.612613	0.855856
8	0.047468	1		0.564346
9	0.047468	1		0.488748
**Average:**	**0.120147**	**1**	**0.711435**	**0.57948**
SE:	0.027092	0	0.073282	0.094462

Fig S2

NORMALIZED TO U87 Alone

Exp#	**U87 Alone**	**THP1 DMSO**	**THP1 JnJ**
1	1	2.2	0.533333
2	1	1.914285714	1.314286
3	1	2.666666667	1
**Average:**	**1**	**2.26031746**	**0.949206**
SE:	0	0.144307506	0.394441
