# Supplementary material for: Microglial-stimulation of glioma invasion involves the EGFR ligand amphiregulin
Source: PLoS One. 2021 Nov 29;16(11):e0260252. doi: 10.1371/journal.pone.0260252 (PMC8629255; doi:10.1371/journal.pone.0260252)

2A AREG.pdf

1067 x 451

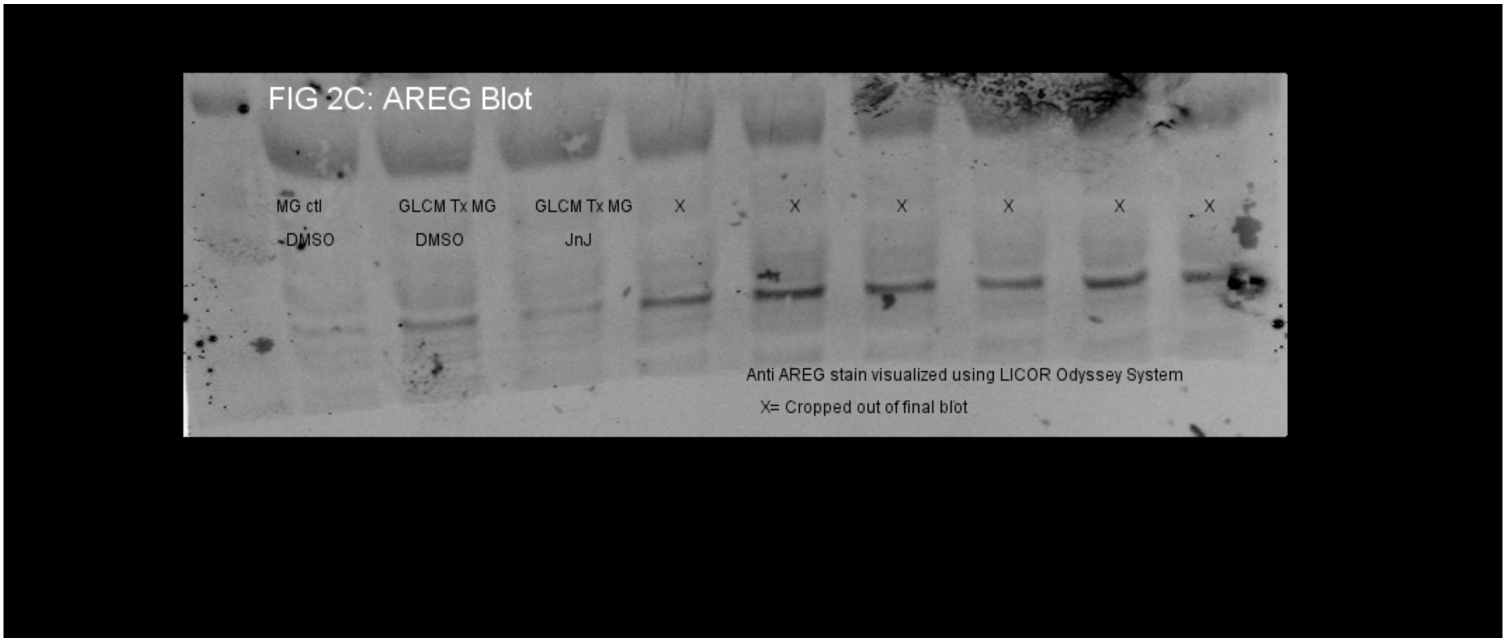

2C Actin.pdf

989 x 332

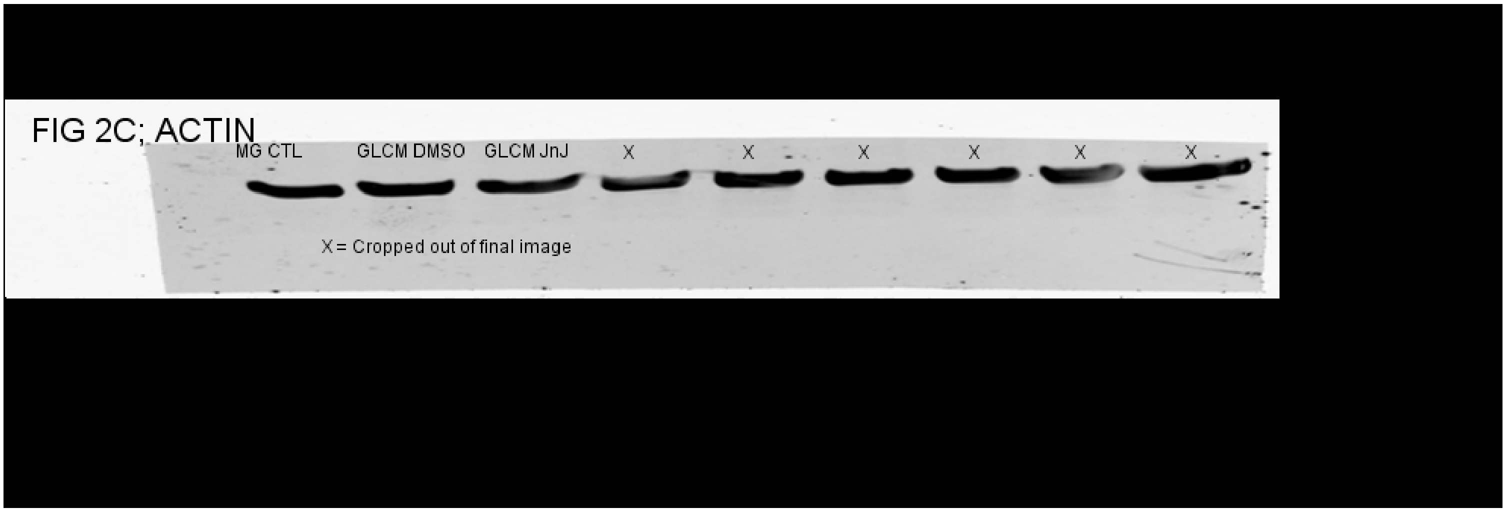

3A AREG.pdf

1046 x 472

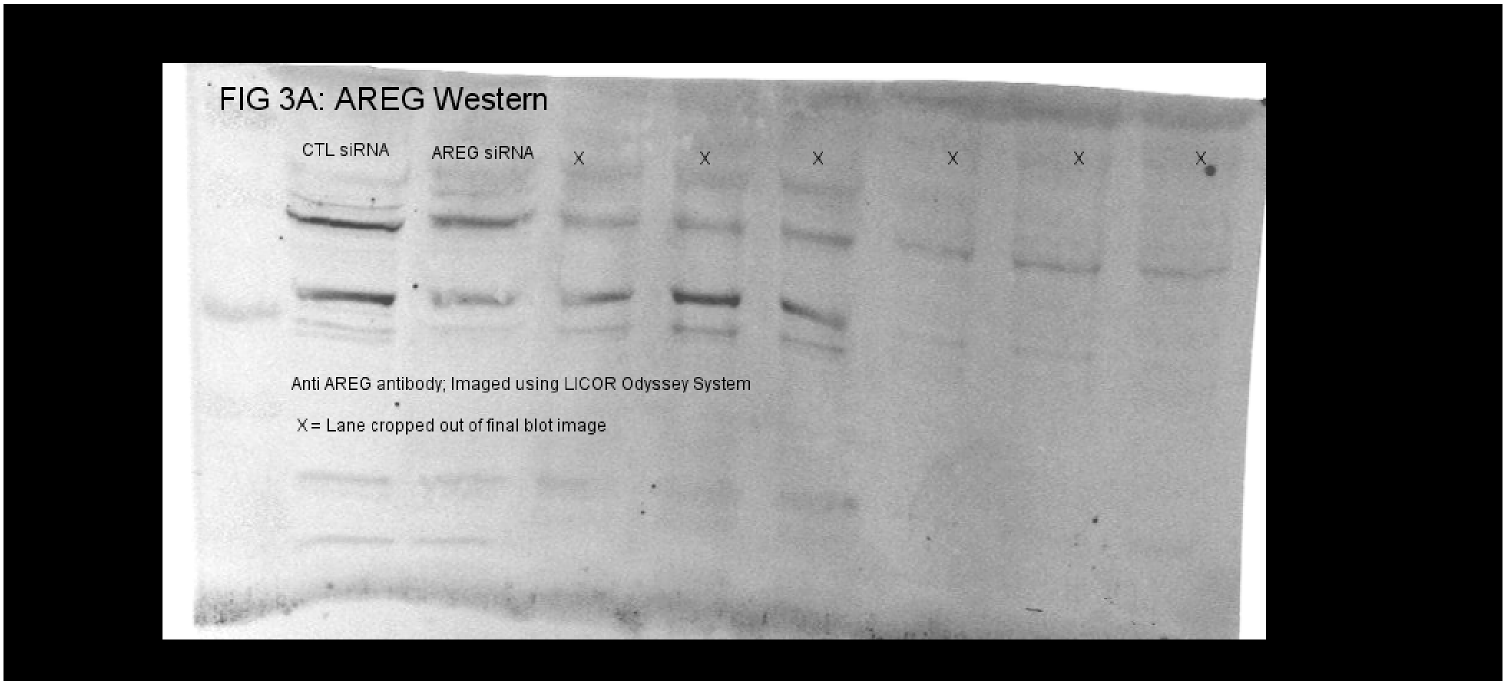

3A Actin.pdf

792 x 78

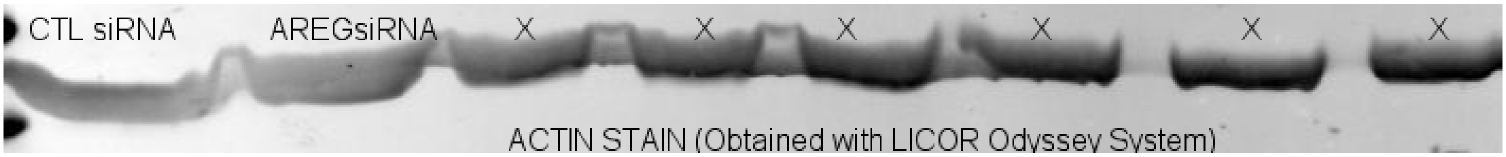

5A ERKp.pdf

1038 x 336

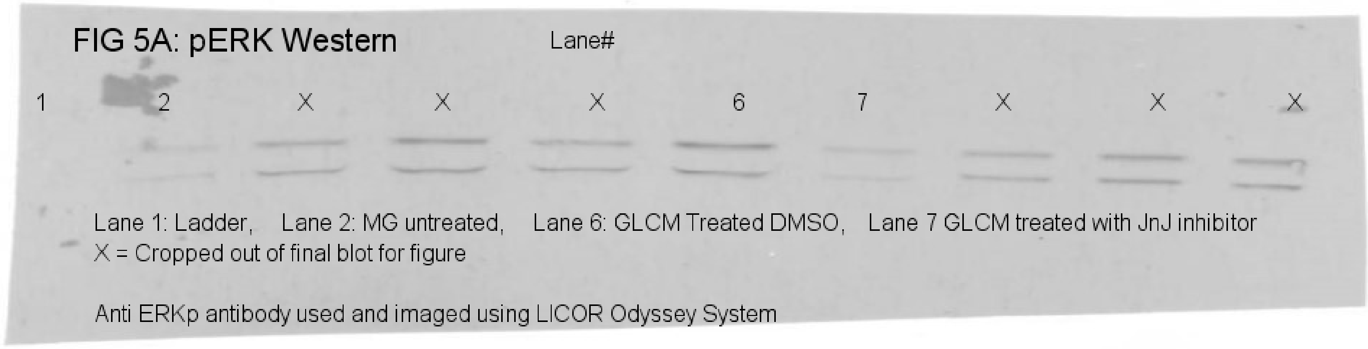

5A Actin.pdf

774 x 258

FIG 5A
ACTIN control

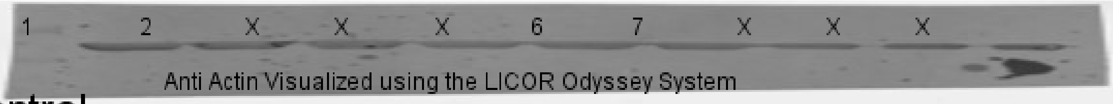

Supplement: S1 Raw images — (PDF) [file pone.0260252.s004.pdf]
