# Supplementary material for: Microglial-stimulation of glioma invasion involves the EGFR ligand amphiregulin
Source: PLoS One. 2021 Nov 29;16(11):e0260252. doi: 10.1371/journal.pone.0260252 (PMC8629255; doi:10.1371/journal.pone.0260252)
Supplement: Attachment — Submitted filename: Reviewer comments - PLOS ONE.docx [file pone.0260252.s005.docx]

**Overall opinion and general observations of the manuscript**

**PONE-D-21-18814**

**Summary**

This is a comprehensive study regarding the mechanisms glioblastoma (GBM) cells use to invade normal brain. It is known that microglia stimulate GBM cell invasion, and that this process is dependent on CSF-1R signaling. However, to have discovered more in-depth mechanism and pro-invasive factors upregulated in microglia, particularly amphiregulin (AREG), is interesting since targeting AREG/EGFR in the tumor could inhibit tumor invasion. Moreover, these extensive results are promising in terms of targeted therapies for GBM.

However, there are some concerns with regard to specific statements.

1. Term "multiforme" is no longer in use. According to the WHO 2016 classification of diffuse gliomas this term is omitted. Moreover, in Abstract you used this term with glioma, which is inappropriate.
2. You have cited plenty of references, however, only less than 20 is issued since 2015. For example, in Introduction you cited 8 references that showed that microglia are enhancers of glioma cell invasion, and none of them is issued since 2015.
3. The last paragraph in Introduction should be the aim of your research, not to conclude what you have found. Otherwise, it looks like Conclusion. This paragraph needs to be changed.
4. I wondered about the concentration of CSF-1R inhibitor (JnJ). Why did you use 10 nM?
5. Can you elaborate the place of inoculation GBM cells? Why did you use those coordinates for induction of glioblastoma? Does it have impact on your model of glioblastoma?
6. Please, indicate magnification you used in Fig.3B and Fig.4. Also, if possible, add scale bar in both microphotographs. Explain what arrows indicate.
7. In Fig.4 the number of invaded cells in group treated with AREG blocking Ab is higher than in U87 alone, while in microphotographs it looks comparable between these two groups. Please, use consistent pictures.
